# Nephrosclerosis-Related Histopathological Findings by Cortical Region From a Japanese Community-Based Study

**DOI:** 10.1016/j.xkme.2025.101194

**Published:** 2025-12-02

**Authors:** Hirokazu Marumoto, Takaya Sasaki, Emi Oishi, Satoko Sakata, Mao Shibata, Yoshihiko Furuta, Jun Hata, Yoshinao Oda, Takanari Kitazono, Nobuo Tsuboi, Takashi Yokoo, Toshiharu Ninomiya

**Affiliations:** 1 Department of Epidemiology and Public Health, Graduate School of Medical Sciences, Kyushu University, Fukuoka, Japan; 2 Department of Medicine and Clinical Science, Graduate School of Medical Sciences, Kyushu University, Fukuoka, Japan; 3 Center for Cohort Studies, Graduate School of Medical Sciences, Kyushu University, Fukuoka, Japan; 4 Department of Anatomic Pathology, Pathological Sciences, Graduate School of Medical Sciences, Kyushu University, Fukuoka, Japan; 5 Division of Nephrology and Hypertension, Department of Internal Medicine, The Jikei University School of Medicine, Tokyo, Japan

**Keywords:** Hypertension, chronic kidney disease, renal pathology

## Abstract

**Rationale & Objective:**

Nephrosclerosis is a major cause of end-stage kidney disease. However, few studies have addressed the association between kidney function and nephrosclerosis-related histopathologic findings because most cases of nephrosclerosis are diagnosed based on clinical signs without a kidney biopsy.

**Study Design:**

Cross-sectional study.

**Setting & Participants:**

Autopsy specimens of kidneys were obtained from 181 individuals who died within 6 years of a community-wide health examination in 2007 and who had an autopsy at the time of death.

**Exposure:**

Histopathologic findings, including glomerular, tubulointerstitial, and vascular lesions, were evaluated as outcome variables in relation to estimated glomerular filtration rate. The kidney cortex in each specimen was divided into 3 equally spaced cortical regions (superficial, middle, and juxtamedullary cortex) to assess depth-dependent lesion distribution.

**Outcomes:**

Glomerular, tubulointerstitial, and vascular lesions were evaluated in cortical regions at different depths.

**Analytical Approach:**

Associations between estimated glomerular filtration rate levels and the extent of histopathologic findings based on cortical region were tested using generalized linear mixed-effects models.

**Results:**

The present study included 172 autopsied cases (mean age: 81 years; men: 50%). The extent of histopathologic lesions progressed significantly with worsening kidney function, and the association was similar in each cortical region. Analysis based on cortical region showed significant gradients in the extent/severity of nephrosclerotic lesions, with global glomerulosclerosis and interstitial fibrosis and tubular atrophy dominating in the superficial cortex and arterial intima-media thickness and arteriolar hyalinosis dominating in the juxtamedullary cortex. The cortical region specificity of histopathologic findings in nephrosclerosis became less prominent with worsening kidney function.

**Limitations:**

Participants were elderly, and the causal relationship between pathologic findings and renal dysfunction could not be determined.

**Conclusions:**

The present study confirms a cortical region--dependent gradient of nephrosclerotic lesions within the kidney and suggests that arterial and tubulointerstitial lesions may be more strongly linked with kidney dysfunction than glomerular lesions.

## Plain-Language Summary

Although nephrosclerosis is a major cause of end-stage renal failure, few studies have addressed its histopathologic relationship with kidney function. In this study, kidney specimens from 172 Japanese community residents at the time of death were evaluated based on 6 histopathologic indices in 3 renal cortical regions. The extent of histopathologic lesions progressed significantly with advancing kidney dysfunction, and the association was similar in each cortical region. The attenuated gradients in tubulointerstitial and arterial lesions, but not glomerular lesions, showed significant associations with kidney dysfunction. Taken together, our present results provide new insights that may enhance our understanding of factors contributing to kidney dysfunction in the general population.

Nephrosclerosis, a disease characterized by sclerosis and fibrosis of the kidney parenchyma due to vascular injury from aging and hypertension,^1^ is one of the major causes of end-stage renal failure worldwide.^2^, ^3^, ^4^ The vessels primarily affected in nephrosclerosis are resistance vessels—that is, the arcuate arteries, interlobular arteries, and afferent arterioles. High blood pressure (BP) and volume overload enlarge the lumina of these vessels and promote hypertrophy and proliferation of intimal smooth muscle cells, along with intimal thickening.^1^^,^^5^, ^6^, ^7^ Arteries in the kidney are distributed from the juxtamedullary cortex to the superficial cortex, gradually thinning and branching,^8^, ^9^, ^10^ and the effects of pressure and capacitive loading can be more extensive at either the center or the periphery. The individual hemodynamics within the kidney strongly influence the extent of pathologic damage. Some animal studies have reported that the superficial cortex at the peripheral end of the arterial system is prone to ischemic injury owing to hypoperfusion^11^^,^^12^; by contrast, the strain-vessel hypothesis^13^ posits that arterioles in the juxtamedullary cortex branching from the proximal arcuate arteries are more susceptible to hyperperfusion injury owing to their having higher BP than the superficial cortical arterioles. However, there are very few histopathologic studies in humans focused on kidney-cortex depth and corresponding kidney function.

In general, kidney tissue for pathologic studies is collected using biopsy, but kidney biopsy is usually performed in patients with marked proteinuria or rapidly progressing kidney dysfunction, not in individuals with normal kidney function.^14^, ^15^, ^16^, ^17^ In addition, it is more difficult to distinguish cortical regions in kidney biopsy specimens compared with autopsy specimens, and thus autopsy specimens are superior for examining potential hemodynamic effects owing to anatomical differences within the kidney. Several previous autopsy series studies have investigated the degree of histopathologic nephrosclerosis according to kidney cortical regions.^18^, ^19^, ^20^, ^21^ However, these studies were conducted on hospitalized patients who died while undergoing treatment, and therefore their results may have been skewed by the influence of severe comorbid conditions or treatments.

The Hisayama study is a prospective study of cardiovascular disease in a Japanese community,^22^^,^^23^ characterized by autopsy verification of the cause of death among approximately 70% of decedents in the population. We, in this study, used autopsy samples from the Hisayama study to determine the association between kidney function and the extent/severity of nephrosclerosis-related histopathologic findings based on cortical region.

## Methods

### Study Population and Autopsy

The Hisayama study was begun in 1961 in Hisayama, a suburb of Fukuoka city on Kyushu Island, Japan.^22^^,^^23^ For the present study, we used autopsy specimens obtained from deceased participants of the Hisayama study. A total of 3,384 residents aged ≥40 years received health examinations at the initial survey from 2007-2008 and were followed up by annual visits. Within 6 years, 292 of these participants died, and 181 of these underwent autopsy and were included in this study. After excluding 3 cases without available kidney specimens, and 6 cases in whom almost all glomeruli showed global glomerular sclerosis or amyloid deposits, the remaining 172 autopsied cases were included in the present study (Fig S1). This study was conducted with the approval of the Kyushu University Institutional Review Board for Clinical Research (2023-74). Written informed consent was obtained from all individuals upon their enrollment in the Hisayama study.

### Histopathologic Assessment of Kidney Tissue and Outcomes

All kidney specimens were fixed in buffered formalin, embedded in paraffin, cut into 2- to 3-μm sections, and stained with hematoxylin and eosin, periodic acid–Schiff, Masson trichrome, and periodic acid–methenamine silver. We obtained images of all cortical areas using light microscopy.

The kidney cortex was identified with reference to the arcuate arteries. The cortical thickness of all patients was measured at 3 locations, and the mean value was calculated. Patients were divided into 2 groups according to the median value of kidney cortical thickness (<4.0 mm vs ≥4.0 mm) for subgroup analysis. The cortex was trisected into 3 equal parts and labeled, in order of proximity to the medulla, as the juxtamedullary cortex, middle cortex, and superficial cortex. We evaluated the following 6 histopathologic findings associated with nephrosclerosis: (1) global glomerulosclerosis, (2) segmental glomerulosclerosis, (3) interstitial fibrosis and tubular atrophy (IFTA), (4) interstitial inflammation, (5) arterial intima-media ratio, and (6) arteriolar hyalinosis index. Glomerular lesions were defined as the percentage of glomeruli with lesions relative to all observed glomeruli. The degree of IFTA and interstitial inflammation was semiquantitatively evaluated by 5% intervals as the percentages of the affected area over each observed cortical area, and the mean values of 3 fields of view at ×40 magnification were calculated. The arterial intima-media ratio was defined as the ratio of intimal thickness to media thickness, and the mean values of 3 arteries were calculated for each cortical area.

Severity of arteriolar hyalinosis was scored as follows: 0, no hyalinosis; 1, partial hyalinosis; 2, hyalinosis covering approximately half of the vessel wall; and 3, hyalinosis covering more than half of the vessel wall and/or hyalinosis covering all layers of the vessels.^18^^,^^24^ In addition, the arteriolar hyalinosis index was defined as the mean arteriolar hyalinosis score for each observed vascular pole.

All histopathologic images were captured by one investigator (HM) with a light microscope (BX43; Olympus) and a digital camera (DP74; Olympus). The captured images were cropped to divide the cortical areas and distributed to the investigators without additional processing or manipulation. Two nephrologists (HM and TS) blinded to the clinical information evaluated all histopathologic findings independently. The 2 investigators’ intraclass correlation coefficients for pathologic findings were 0.54 and 0.93 (Table S1). The mean value of the measurements of the 2 investigators was used for analysis.

### Measurement of Risk Factors

At the health examinations in 2007 and 2008, self-administered questionnaires containing baseline information on smoking habits were completed by each participant and confirmed by well-trained interviewers. Body height and weight were measured in light clothing without shoes, and body mass index was calculated as body weight in kilograms divided by body height in meters squared (kg/m^2^). BP was measured 3 times using an automatic sphygmomanometer with the participant in a seated position after at least 5 minutes of rest. The average of the 3 measurements was used for analysis. Hypertension was defined as systolic BP ≥ 140 mm Hg, diastolic BP ≥ 90 mm Hg, or the current use of antihypertensive medications. Diabetes mellitus was determined based on plasma glucose levels measured using the hexokinase method at the health examination (fasting glucose level ≥ 7.0 mmol/L or postprandial glucose level ≥ 11.1 mmol/L) or based on the current use of oral glucose-lowering medications or insulin.^25^ Hypercholesterolemia was defined as serum total cholesterol ≥ 5.69 mmol/L or the current use of lipid-modifying agents. Proteinuria was defined as 1+ (equivalent to a urinary protein-creatinine ratio of 0.15-0.49 g/g in quantitative tests)^26^ or greater by the urine dipstick test. The estimated glomerular filtration rate (eGFR) was calculated using the creatinine-based Chronic Kidney Disease Epidemiology Collaboration equation, modified by the Japanese coefficient,^27^ with serum creatinine levels measured enzymatically. Kidney dysfunction was defined as eGFR < 60 mL/min/1.73 m^2^.

### Statistical Analysis

Data were expressed as mean values, median values, or frequencies. Trends in the mean values or the frequencies of risk factors across eGFR categories were determined using linear regression analysis and the Cochran-Armitage test, respectively. The Jonckheere-Terpstra test was used to examine trends among the median values of histopathologic findings of overall cortical regions, eGFR categories, and tertile groups of the arteriolar hyalinosis index and arterial intima-media ratios. The Friedman test was used to examine trends in median values of histopathologic extent/severity based on cortical region. Associations between eGFR levels and the extent of histopathologic findings based on cortical region were tested using generalized linear mixed-effects models with random intercept and random slope for unadjusted, age- and sex-adjusted, and multivariable-adjusted models. In the multivariable-adjusted analysis, the estimates were adjusted for potential risk factors for kidney impairment—namely, age, sex, diabetes mellitus, hypertension, hypercholesterolemia (1 missing value), serum uric acid, body mass index, and current smoking. The following 3 categories of kidney function were defined: (1) eGFR ≥ 60 mL/min/1.73 m^2^, (2) eGFR 45 to <60 mL/min/1.73 m^2^, and (3) eGFR < 45 mL/min/1.73 m^2^. Because segmental glomerulosclerosis, IFTA, stromal inflammatory cell infiltration, and the arterial intima-media ratio had skewed distributions, we used a general linear model after transforming them to natural logarithms. For segmental glomerulosclerosis, 0.5 was added to each value before transformation to account for cases with a value of 0. To clarify the distribution of lesions based on cortical region, we defined the superficial-to-juxtamedullary cortex ratio as the value for the superficial cortex divided by that for the juxtamedullary cortex in each case; this value was then transformed using the ordinary logarithm. Pearson correlation coefficient was calculated to examine the correlation between the continuous variable of eGFR and each pathologic finding or the superficial-to-juxtamedullary cortex ratio in each case. Among the findings in the whole kidney cortex, the intima-media ratio and the arteriolar hyalinosis index were divided into tertiles designated as T1, T2, and T3 in the ascending order (arteriolar hyalinosis index: T1, <0.587; T2, 0.587 to <0.823; T3, ≥0.823; arterial intima-media ratio: T1, <0.832; T2, 0.832 to <1.043; T3, ≥1.043). All statistical analyses were performed using SAS version 9.4 (SAS Institute Inc). Two-sided *P* values < 0.05 were considered statistically significant in all analyses.

## Results

### Population Characteristics and Histopathologic Findings Based on eGFR Levels

**Figure 1:**
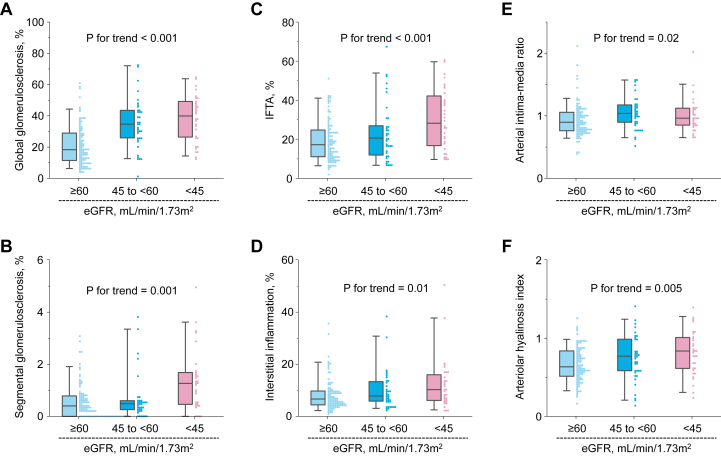
Histopathologic scores according to eGFR levels. Scores for (A) global glomerulosclerosis, (B) segmental glomerulosclerosis, (C) IFTA, (D) interstitial inflammation, (E) arterial intima-media ratio, and (F) arteriolar hyalinosis index are shown according to the eGFR levels (n = 172). Data are shown as box-and-whisker plots and bee swarm plots. Abbreviations: eGFR, estimated glomerular filtration rate; IFTA, interstitial fibrosis and tubular atrophy.

Among the 172 autopsied cases, the mean age at death was 80.5 years, and the proportion of men was 50.0% (Table 1). The mean period from the last examination to death was 3.1 years. A lower eGFR level was associated with older age, serum uric acid levels, and the proportions of individuals using antihypertensive agents or otherwise identified as hypertensive. With regard to histopathology, the degree of all the histopathologic findings—that is, global glomerulosclerosis, segmental glomerulosclerosis, IFTA, interstitial inflammation, arterial intima-media ratio, and arteriolar hyalinosis index—in all kidney cortical regions increased significantly as eGFR levels decreased (Fig 1).

**Table 1:** Mean Values or Frequencies of Potential Risk Factors and Laboratory Variables According to eGFR Levels for 172 Autopsied Cases

Risk Factors	All Cases (n = 172)	eGFR Levels			
		≥60 mL/min/1.73 m^2^ (n = 103)	45 to <60 mL/min/1.73 m^2^ (n = 37)	<45 mL/min/1.73 m^2^ (n = 32)	*P* for Trend
Age at death, y	80.5	77.3	86.3	84.2	<0.001
Male, n (%)	86 (50.0)	55 (53.4)	21 (56.8)	10 (31.3)	0.06
Systolic blood pressure, mm Hg	133.6	131.4	142.9	129.6	0.68
Diastolic blood pressure, mm Hg	75.5	75.7	78.9	70.8	0.15
Use of antihypertensive agents, n (%)^a^	80 (46.8)	40 (39.2)	21 (56.8)	19 (59.4)	0.02
Hypertension, n (%)	102 (59.3)	52 (50.5)	27 (73.0)	23 (71.9)	0.01
Diabetes mellitus, n (%)	36 (20.9)	22 (21.4)	9 (24.3)	5 (15.6)	0.61
Hypercholesterolemia, n (%)^a^	50 (29.1)	29 (28.2)	8 (21.6)	13 (40.6)	0.32
Use of lipid-modifying agents, n (%)^a^	28 (16.4)	16 (15.7)	5 (13.5)	7 (21.9)	0.52
Serum uric acid, μmol/L	315	286	321	393	<0.001
Proteinuria, n (%)^b^	34 (22.7)	14 (15.8)	8 (22.9)	12 (41.4)	0.004
Body mass index, kg/m^2^	21.2	20.9	22.0	21.4	0.31
Smoking habits, n (%)	16 (9.3)	10 (9.7)	3 (8.1)	3 (9.4)	0.89

*Note:* Data are shown as mean values or percentages.

Abbreviation: eGFR, estimated glomerular filtration rate.

a One missing value.

b Twenty-two missing values.

### Histopathologic Findings Based on Cortical Regions

**Figure 2:**
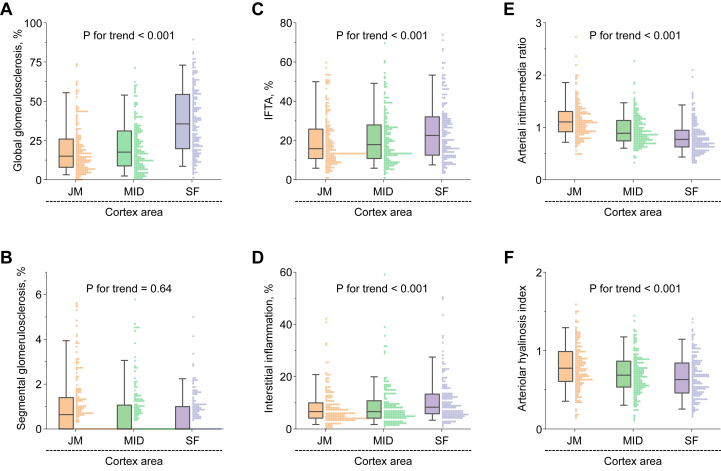
Histopathologic scores according to kidney cortex regions. Scores for (A) global glomerulosclerosis, (B) segmental glomerulosclerosis, (C) IFTA, (D) interstitial inflammation, (E) arterial intima-media ratio, and (F) arteriolar hyalinosis index are shown according to the kidney cortex regions (n = 172). Data are shown as box-and-whisker plots and bee swarm plots. Abbreviations: IFTA, interstitial fibrosis and tubular atrophy; JM, juxtamedullary; MID, middle; SF, superficial.

Next, we compared the degree of histopathologic lesions across 3 kidney cortical regions. As shown in Fig 2, the degree of global glomerulosclerosis, IFTA, and interstitial inflammation increased significantly from the deep to superficial layers of the kidney cortex; that is, juxtamedullary cortex < middle cortex < superficial cortex. Conversely, the arterial intima-media ratio and the arteriolar hyalinosis index decreased significantly from the deep to superficial regions. There was no evidence of significant association between the degree of segmental glomerulosclerosis and the cortical regions. These associations remained consistent after adjusting for potential confounding factors (Table S2).

### Histopathologic Findings Based on Cortical Regions With or Without Kidney Dysfunction

**Figure 3:**
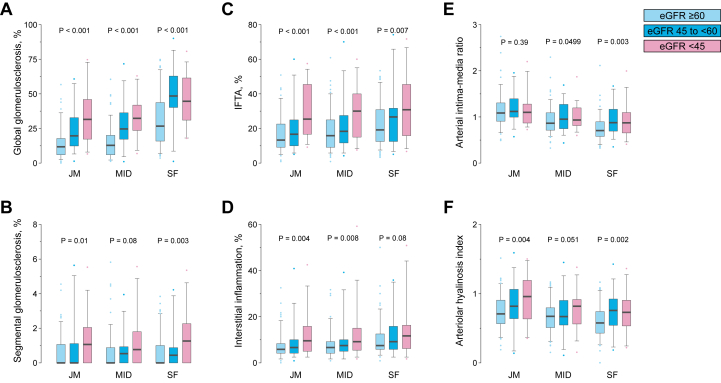
Histopathologic scores according to both cortex region and eGFR. Scores for (A) global glomerulosclerosis, (B) segmental glomerulosclerosis, (C) interstitial fibrosis and tubular atrophy, (D) interstitial inflammation, (E) arterial intima-media ratio, and (F) arteriolar hyalinosis index are shown according to the kidney cortex regions with consideration for eGFR levels (n = 172). Data are shown as a box-and-whisker plot. Abbreviations: eGFR, estimated glomerular filtration rate; JM, juxtamedullary; MID, middle; SF, superficial.

**Figure 4:**
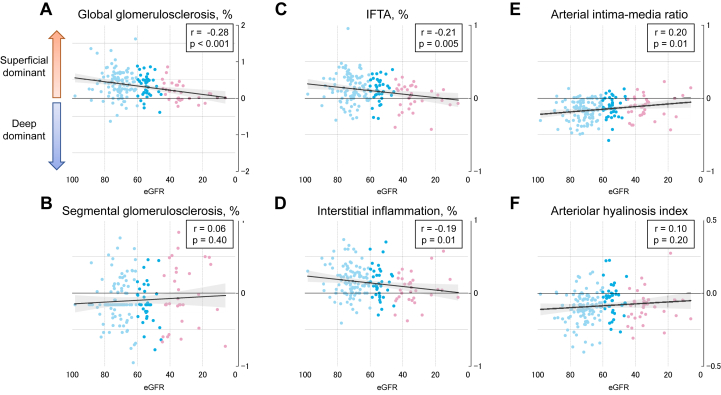
Correlation between the eGFR levels and the ratio of histopathologic scores in the superficial cortex compared with those in the juxtamedullary cortex (n = 172). Correlations between the eGFR levels and the ratio of scores for (A) global glomerulosclerosis, (B) segmental glomerulosclerosis, (C) IFTA, (D) interstitial inflammation, (E) arterial intima-media ratio, and (F) arteriolar hyalinosis index are shown. The solid lines indicate regression lines. Plots are color coded for the 3 groups of chronic kidney disease G1-2, G3a, and G3b-G5. Abbreviations: eGFR, estimated glomerular filtration rate; IFTA, interstitial fibrosis and tubular atrophy.

In addition, we investigated the association between eGFR levels and the extent/severity of each histopathologic finding according to kidney cortical regions. In all the kidney cortical regions, lower eGFR levels were significantly or marginally associated with an increased extent of the following 5 histopathologic findings: (1) global glomerulosclerosis, (2) segmental glomerulosclerosis, (3) IFTA, (4) interstitial inflammation, and (5) arteriolar hyalinosis index (Fig 3). In the middle and superficial cortical regions, but not the juxtamedullary region, a lower eGFR level was also significantly associated with the severity of the arterial intima-media ratio. When examining the correlation between eGFR and the severity of each pathologic finding across cortical regions, significant correlations were observed for all pathologic findings in all cortical regions except for interstitial inflammation in the superficial cortex (Fig S2). As shown in Table 2, we calculated the β value of eGFR for each cortical region with multivariate analysis using each pathologic finding as the objective variable and examined the interaction effect. The model adjusted for age and sex showed significant results with respect to the arterial intima-media ratio, but after multivariate adjustment, there was no interaction between any of the pathologic findings and cortical region. To further visualize the correlation between kidney function and histopathologic findings based on cortical region, we drew scatter plots between the eGFR levels and the superficial-to-juxtamedullary cortex ratio for each histopathologic finding (Fig 4). A lower eGFR level was significantly associated with an increased superficial-to-juxtamedullary cortex ratio of the intima-media ratio and with a decreased superficial-to-juxtamedullary cortex ratio of global glomerulosclerosis, IFTA, and interstitial inflammation.

**Table 2:** Extent/Severity of Histopathologic Lesions Based on Cortical Region Per 1-SD Decrease in eGFR

Cortical Region	Age and Sex Adjusted				Multivariable Adjusted^a^			
	*β* (95% CI) for Each 1-SD Decrease in eGFR		*P* Value	*P* for Interaction	*β* (95% CI) for Each 1-SD Decrease in eGFR		*P* Value	*P* for interaction
Global glomerulosclerosis, %								
JM	8.57	(6.31-10.84)	<0.001	0.22	9.18	(6.69-11.67)	<0.001	0.18
MID	6.96	(4.66-9.25)	<0.001		7.17	(4.66-9.67)	<0.001	
SF	5.81	(2.54-9.09)	<0.001		6.04	(2.43-9.65)	0.001	
Segmental glomerulosclerosis, %								
JM	0.21	(0.09-0.34)	<0.001	0.76	0.22	(0.07-0.36)	0.003	0.88
MID	0.20	(0.08-0.32)	0.01		0.14	(0.01-0.27)	0.04	
SF	0.24	(0.12-0.37)	<0.001		0.21	(0.07-0.34)	0.004	
IFTA, %								
JM	0.27	(0.16-0.38)	<0.001	0.18	0.31	(0.19-0.43)	<0.001	0.15
MID	0.20	(0.09-0.32)	<0.001		0.26	(0.14-0.39)	<0.001	
SF	0.15	(0.04-0.26)	0.006		0.20	(0.08-0.32)	0.001	
Interstitial inflammation, %								
JM	0.20	(0.08-0.33)	<0.001	0.25	0.23	(0.10-0.37)	0.001	0.21
MID	0.19	(0.06-0.31)	0.004		0.24	(0.10-0.38)	0.001	
SF	0.11	(-0.01-0.23)	0.06		0.17	(0.04-0.30)	0.01	
Arterial intima-media ratio								
JM	0.01	(-0.04 to 0.06)	0.64	0.04	0.03	(-0.03 to 0.08)	0.38	0.05
MID	0.04	(-0.01 to 0.09)	0.11		0.05	(-0.01 to 0.11)	0.1	
SF	0.06	(0.00-0.12)	0.03		0.08	(0.01-0.14)	0.02	
Arteriolar hyalinosis index								
JM	0.08	(0.03-0.13)	0.001	0.93	0.08	(0.03-0.14)	0.002	0.86
MID	0.04	(0.00-0.09)	0.05		0.06	(0.01-0.10)	0.02	
SF	0.07	(0.03-0.12)	0.001		0.07	(0.02-0.12)	0.005	

Abbreviations: CI, confidence interval; eGFR, estimated glomerular filtration rate; IFTA, interstitial fibrosis and tubular atrophy; JM, juxtamedullary cortex; LS, least squares; MID, middle; SD, standard deviation; SF, superficial.

a Adjusted for age, sex, systolic blood pressure, use of antihypertensive agents, diabetes mellitus, hypercholesterolemia, serum uric acid, body mass index, and smoking habits.

### Association Between Arteriosclerosis and Global Glomerulosclerosis

**Figure 5:**
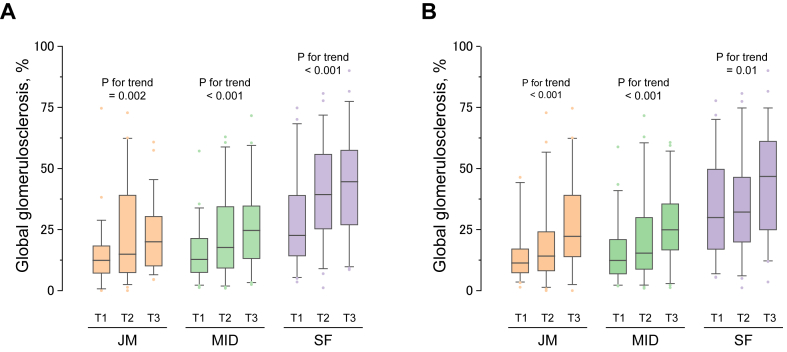
Association between the prevalence/severity of vascular lesions in the kidney cortex and the proportion of global glomerulosclerosis based on kidney cortex region (n = 172). In global glomerulosclerosis, each cortical area is shown to be divided into tertiles of (A) intima-media ratio and (B) arteriolar hyalinosis index calculated from the means of the total kidney areas. The tertiles of the arterial intima-media ratio were as follows: T1, <0.832; T2, 0.832 to <1.043; T3, ≥1.043. The tertiles of the arteriolar hyalinosis index were as follows: T1, <0.587; T2, 0.587 to <0.823; T3, ≥0.823. Abbreviations: JM, juxtamedullary; MID, middle; SF, superficial.

We examined the association of arteriosclerotic lesions with global glomerulosclerosis with vascular lesions based on each kidney cortex region (Fig 5). Higher tertile levels of the intima-media ratio and the arteriolar hyalinosis index were associated with a significantly greater extent of global glomerulosclerosis in each cortical region.

### Subgroup Analyses Stratified Based on Clinical Risk Factors

Finally, we performed subgroup analyses stratified based on clinical risk factors such as age, diabetes, hypertension, cortical thickness, and proteinuria to assess whether these factors modify the association between cortical pathology and region (juxtamedullary, middle, and superficial) (Table S3). All cortical regions showed more severe pathologic damage in patients with high-risk profiles, including patients with older age, diabetes, hypertension, and proteinuria. Significant interactions were observed only in segmental sclerosis and IFTA.

## Discussion

The present study evaluated differences in the magnitude of the association between kidney function and the severity of histopathologic findings of nephrosclerotic lesions across different cortical regions using autopsied kidneys from an ongoing prospective cohort study of Japanese community residents. Lower eGFR levels were significantly linked to increased severity of histopathologic lesions in all cortical regions, with the superficial cortex showing more severe lesions of most kinds than the deeper regions. Analysis of cortical depths revealed variations in lesion distribution, independent of confounders, which was as follows: global glomerulosclerosis, IFTA, and interstitial inflammation were predominant in the superficial cortex, whereas arteriosclerotic lesions prevailed in the juxtamedullary cortex. Subgroups with kidney dysfunction displayed weaker cortical region gradients of IFTA, interstitial inflammation, and arterial intima-media ratio compared with those without kidney dysfunction. Moreover, arteriosclerotic lesions across the cortex were positively associated with global glomerulosclerosis. Although this is an observational study and causality cannot be precisely stated, these findings align with the current understanding of the progression of chronic kidney disease and may offer new insights into the cortical distribution of histopathologic lesions, enhancing understanding of the disease progression.

To estimate regional differences in nephrosclerosis, pathologic evaluation of the kidney is essential, with either kidney biopsy or autopsy. Biopsy is typically only performed in cases with urinary findings or progressive renal damage and may not give an accurate snapshot of the whole cortex; on the other hand, most autopsy series, despite respectively finding that global glomerulosclerosis is predominant in the superficial cortex and arteriolar hyalinosis in the juxtamedullary cortex,^18^, ^19^, ^20^, ^21^ have been hospital based and hence subject to biases, such as the presence of underlying diseases. In this regard, our present study, using a large number of autopsies from the general population, is best suited to accurately identify the histopathologic lesions associated with nephrosclerosis and their distribution within the kidney.

The present study also showed a significantly weaker association between the cortical depths and severity of some histopathologic findings (ie, IFTA, interstitial inflammation, and arterial intima-media ratio) in relation to the decline in eGFR. In addition, we found that the normal logarithm of the superficial-to-juxtamedullary cortex ratio of these histopathologic findings approached 0 as the eGFR levels decreased. These findings suggest that, as kidney dysfunction progresses, the cortical region gradients of severity of certain histopathologic findings decrease. Our result of attenuated cortical region gradients in some histopathologic findings, including atherosclerotic lesions and tubulointerstitial lesions, in cases with kidney dysfunction may suggest that these lesions play a more prominent role in the progression of nephrosclerosis compared with glomerular lesions.

According to the “Strain vessels hypothesis,” the afferent arteriole in the juxtamedullary cortex, branching directly from relatively large vessels, receives high pulse pressure from the arcuate artery.^8^, ^9^, ^10^^,^^13^ As a result, glomeruli in the juxtamedullary cortex are more susceptible to hypertensive kidney damage than those in the superficial cortex. In addition to anatomical differences, physiological differences exist between the glomeruli of the juxtamedullary and superficial cortex. Although the overall oxygen demand of the kidney is high, the partial pressure of oxygen in the juxtamedullary cortex is relatively low compared with that in the superficial cortex, which may also contribute to the reduced efficiency of the glomeruli of the juxtamedullary cortex.^28^ The greater intima-media thickness and arteriolar hyalinosis in the juxtamedullary cortex observed in the present study were consistent with this hypothesis. The peritubular capillaries arise from the efferent arteries and play an essential role in supplying oxygen and nutrients to the tubules and interstitial cells,^1^^,^^29^ and they are considered pivotal in the final common pathway of kidney disease progression.^30^, ^31^, ^32^ Therefore, alterations in upstream glomerular hemodynamics can cause direct tubulointerstitial injury and subsequently global glomerulosclerosis. In this study, advanced intima-media thickness and arteriolar hyalinosis were also associated with global glomerulosclerosis, which was predominant in the superficial cortex. Our finding that atherosclerotic lesions become more advanced from the deep to the superficial layers may suggest that the progression of atherosclerotic lesions in the kidney is followed by glomerulosclerosis and possibly tubulointerstitial lesions. After multivariable adjustment, the arterial intima-media ratios in the middle and deep cortex were not significantly associated with eGFR. This may reflect the role of the arterial intima-media ratio in the middle and deep cortex as a marker of chronic vascular remodeling or systemic vascular aging—pathologic processes that do not necessarily compromise renal perfusion or function, particularly in older individuals with preserved autoregulation. Further studies are needed to elucidate the sequential changes in histopathology that may arise during the progression of nephrosclerosis.

Further segmentation of kidney cortex based on cortical thickness was proposed by Denic et al,^20^ who divided thick wedge sections into 5 depth regions. They reported that a thinner kidney cortex was strongly associated with glomerular sclerosis, particularly in superficial glomeruli. In our study, consistent with their results, more severe lesions were observed in the thinner cortices, with significant interactions in global glomerulosclerosis and IFTA. Importantly, the associations between kidney cortical regions and histopathologic findings remained significant regardless of cortical thickness. From the above, differences in cortical thickness do not negate the association between kidney cortical regions and histopathologic findings per se. However, more segmentation of kidney cortical regions may provide a more complete picture of the spatial distribution of microscopic lesions within the kidney cortex and their relationship with kidney dysfunction, and a more precise understanding of pathologic progression. Future studies with more subdivided cortical regions are expected to confirm their results.

Consistent with clinical expectations, patients with high-risk profiles—such as older age, diabetes, hypertension, and proteinuria—showed more severe pathologic findings across all cortical regions. However, significant interactions were observed only for age in relation to segmental sclerosis and IFTA. In these cases, the typically juxtamedullary dominant pattern for segmental sclerosis and the superficial dominant pattern for IFTA appeared to be attenuated in older individuals. Given that our study included autopsy cases with an advanced median age of 80.5 years, these interactions may have been due to chance or may reflect age-related diffuse injury across the kidney cortex that obscures regional differences.

This study’s main strength was the use of autopsied cases from a community-based cohort study with high autopsy rates. We evaluated many more glomeruli per case than are typically obtained in kidney biopsy specimens. However, several limitations should be noted. First, the eGFR levels may have changed drastically during the 6-year (max) interval (average, 3.1 years) between the last recorded health examination and the autopsy, which could have influenced our results regarding the correlation between renal function and histological findings. Therefore, we evaluated whether the cortical distribution of lesions varied by time interval and found consistent patterns across kidney cortical regions, except for modest interactions in global glomerulosclerosis and segmental sclerosis (Table S2). Therefore, although the interval length had some influence on the strength of the associations, it is unlikely to have undermined the associations observed, or to have altered our conclusions regarding the lesion distribution. Second, the findings, despite being community based, may not be generalizable to general populations because most autopsied cases were older people. Third, the present cross-sectional study cannot address the causal relationship between nephrosclerotic lesions or their expansions across the kidney and the development of kidney dysfunction. Fourth, the histopathologic evaluations were performed by nephrologists rather than by renal pathologists. However, both evaluators received prior training, and the assessed lesions demonstrated moderate-to-excellent interobserver agreement, with intraclass correlation coeofficients ranging from 0.54-0.93 (Table S1). Although the possibility of some misclassification cannot be entirely ruled out, its impact on the overall findings is likely to be minimal. Fifth, although primary glomerulonephritis could not be completely excluded owing to the nature of autopsy-based sampling, the absence of proliferative changes or glomerular basement membrane abnormalities on light microscopy argues against significant glomerular pathology. Given the very low estimated prevalence of primary glomerular diseases in the general population (<0.01%)^33^ and the consistent results across subgroups based on diabetes, a major secondary cause of kidney disease, we believe that the potential influence of primary or secondary glomerular diseases on our findings was minimal. Finally, we fully acknowledge that estimating the exact percentage of involvement can be inherently subjective, particularly in the intermediate ranges (eg, distinguishing 10% from 15%). However, we should emphasize that the semiquantitative scoring was conducted by 2 experienced nephrologists who independently assessed each specimen, and interobserver agreement was good for both inflammation and IFTA, as shown in Table S1. Moreover, to test the robustness of our findings, we conducted an additional sensitivity analysis using categorized thresholds (ie, 5%, 25%, and 50%), and the trends remained consistent with the main analysis (Tables S4 and S5). The nonsignificant trend in the analysis using 5% for the IFTA cutoff may have been partly due to the very limited number of subjects having IFTA of <5%. Thus, these findings support the validity of our semiquantitative approach.

In conclusion, we used autopsy kidney specimens from a community-based cohort study to investigate histopathologic findings related to nephrosclerosis, with consideration for kidney function and cortical region depth. The cortical region gradients identified in some histopathologic findings were attenuated with decreasing kidney function. Taken together, our results may reflect potential associations between these types of histological lesions and nephrosclerosis progression and may contribute to a better understanding of the pathophysiology of kidney dysfunction.
