# Supplementary material for: Nephrosclerosis-Related Histopathological Findings by Cortical Region From a Japanese Community-Based Study
Source: Kidney Med. 2025 Dec 2;8(2):101194. doi: 10.1016/j.xkme.2025.101194 (PMC12859235; doi:10.1016/j.xkme.2025.101194)
Supplement: Supplementary File (PDF) — Figures S1 and S2; Tables S1-S5. [file mmc1.pdf]

Table S1. Intraclass correlation coefficients for pathological findings.

	ICC
**Global glomerulosclerosis**	0.927
**Segmental glomerulosclerosis**	0.543
**IFTA**	0.932
**Interstitial inflammation**	0.887
**Arterial intima-media ratio**	0.844
**Arteriolar hyalinosis index**	0.918

Abbreviations: ICC, Intraclass correlation coefficients; IFTA, interstitial fibrosis/tubular atrophy.

Table S2. The extent/severity of histopathological lesions by cortical region after multivariable adjustment.

	Age- and sex- adjusted				Multivariable-adjusted			
	Adjusted mean values (95% CI)		P value	P for trend	Adjusted mean values (95% CI)		P value	P for trend
**Global glomerulosclerosis, %**								
JM	19.6	(17.1 to 22.1)	Ref		21.3	(17.0 to 25.5)	Ref	
MID	21.2	(18.7 to 23.7)	0.15	<0.001	22.6	(18.4 to 26.9)	0.21	<0.001
SF	37.5	(35.0 to 40.0)	<0.001		38.9	(34.6 to 43.1)	<0.001	
**Segmental glomerulosclerosis, %**								
JM	0.92	(0.83 to 1.03)	Ref		0.94	(0.79 to 1.12)	Ref	
MID	0.91	(0.82 to 1.02)	0.84	0.56	0.92	(0.77 to 1.09)	0.71	0.67
SF	0.96	(0.86 to 1.07)	0.43		0.97	(0.82 to 1.15)	0.43	
**IFTA, %**								
JM	16.3	(14.7 to 18.0)	Ref		19.5	(16.3 to 23.6)	Ref	
MID	17.5	(15.8 to 19.3)	0.02	<0.001	20.9	(17.5 to 25.0)	0.02	<0.001
SF	20.9	(18.9 to 23.1)	<0.001		25.0	(20.9 to 30.0)	<0.001	
**Interstitial inflammation, %**								
JM	6.4	(5.8 to 7.1)	Ref		7.2	(5.9 to 8.8)	Ref	
MID	6.8	(6.1 to 7.6)	0.04	<0.001	7.7	(6.3 to 9.4)	0.03	<0.001
SF	8.8	(7.9 to 9.8)	<0.001		10.0	(8.2 to 12.2)	<0.001	
**Arterial intima-media ratio**								
JM	1.09	(1.04 to 1.14)	Ref		1.12	(1.03 to 1.21)	Ref	
MID	0.91	(0.87 to 0.95)	<0.001	<0.001	0.93	(0.86 to 1.01)	<0.001	<0.001
SF	0.77	(0.73 to 0.81)	<0.001		0.79	(0.73 to 0.85)	<0.001	
**Arteriolar hyalinosis index**								
JM	0.79	(0.75 to 0.83)	Ref		0.74	(0.67 to 0.82)	Ref	
MID	0.70	(0.66 to 0.74)	<0.001	<0.001	0.65	(0.57 to 0.72)	<0.001	<0.001
SF	0.66	(0.62 to 0.70)	<0.001		0.61	(0.53 to 0.68)	<0.001	

Abbreviations: IFTA, interstitial fibrosis/tubular atrophy; JM, juxtamedullary cortex; MID, middle cortex; SF, superficial cortex.

Table S3. Interaction histological findings in cortical regions to each risk factors.

	Last examination to death			cortical thickness			Aging			Diabetes mellitus			Hypertension			Proteinuria		
	Adjusted mean values (95% CI)		P for interaction	Adjusted mean values (95% CI)		P for interaction	Adjusted mean values (95% CI)		P for interaction	Adjusted mean values (95% CI)		P for interaction	Adjusted mean values (95% CI)		P for interaction	Adjusted mean values (95% CI)		P for interaction
	≥3y	<3y		Thin	Thick		<80.5y	≥80.5y		No	Yes		No	Yes		No	Yes	
**Global glomerulosclerosis, %**																		
JM	22.5<br>(18.8 to 26.3)	16.8<br>(13.6 to 20.0)	0.02	22.9<br>(19.4 to 26.4)	16.3<br>(13.1 to 19.5)	0.001	15.6<br>(12.3 to 19.0)	23.6<br>(20 to 27.2)	0.12	19.3<br>(16.6 to 21.9)	20.9<br>(15.1 to 26.8)	0.79	17.3<br>(13.9 to 20.8)	21.2<br>(17.9 to 24.4)	0.13	17.4<br>(14.6 to 20.3)	26.3<br>(20.2 to 32.4)	0.97
MID	23.2<br>(19.5 to 27.0)	19.3<br>(16.1 to 22.5)		24.4<br>(20.9 to 28)	18.0<br>(14.7 to 21.2)		16.5<br>(13.1 to 19.8)	25.9<br>(22.3 to 29.5)		20.8<br>(18.1 to 23.5)	22.7<br>(16.8 to 28.6)		18.0<br>(14.5 to 21.4)	23.4<br>(20.1 to 26.7)		18.9<br>(16.1 to 21.7)	28.9<br>(22.8 to 35)	
SF	37.5<br>(33.8 to 41.3)	37.5<br>(34.3 to 40.7)		44.9<br>(41.4 to 48.4)	30.1<br>(26.9 to 33.3)		31.7<br>(28.3 to 35.1)	43.3<br>(39.7 to 46.9)		37.3<br>(34.7 to 40.0)	38.2<br>(32.3 to 44.1)		33.1<br>(29.6 to 36.6)	40.5<br>(37.2 to 43.8)		34.9<br>(32.1 to 37.8)	43.7<br>(37.6 to 49.8)	
**Segmental glomerulosclerosis, %**																		
JM	-0.07<br>(-0.23 to 0.08)	-0.08<br>(-0.23 to 0.07)	0.048	-0.04<br>(-0.19 to 0.11)	-0.11<br>(-0.27 to 0.04)	0.44	-0.17<br>(-0.32 to -0.02)	0.02<br>(-0.14 to 0.18)	0.03	-0.07<br>(-0.19 to 0.05)	-0.10<br>(-0.33 to 0.13)	0.77	-0.21<br>(-0.37 to -0.05)	0.02<br>(-0.13 to 0.16)	0.95	-0.12<br>(-0.26 to 0.01)	-0.06<br>(-0.29 to 0.17)	1.00
MID	-0.09<br>(-0.25 to 0.06)	-0.09<br>(-0.24 to 0.07)		-0.14<br>(-0.29 to 0.01)	-0.04<br>(-0.19 to 0.11)		-0.11<br>(-0.26 to 0.03)	-0.07<br>(-0.22 to 0.09)		-0.09<br>(-0.21 to 0.04)	-0.11<br>(-0.34 to 0.13)		-0.16<br>(-0.32 to 0.00)	-0.04<br>(-0.19 to 0.11)		-0.10<br>(-0.23 to 0.04)	-0.02<br>(-0.25 to 0.21)	
SF	0.03<br>(-0.13 to 0.18)	-0.10<br>(-0.26 to 0.05)		-0.12<br>(-0.27 to 0.04)	0.04<br>(-0.11 to 0.19)		-0.08<br>(-0.23 to 0.07)	0.00<br>(-0.16 to 0.16)		-0.04<br>(-0.17 to 0.08)	-0.02<br>(-0.25 to 0.22)		-0.16<br>(-0.31 to 0.00)	0.04<br>(-0.10 to 0.19)		0.02<br>(-0.12 to 0.15)	-0.05<br>(-0.28 to 0.18)	
**IFTA, %***																		
JM	2.88<br>(2.74 to 3.02)	2.70<br>(2.57 to 2.84)	0.22	2.88<br>(2.74 to 3.03)	2.70<br>(2.56 to 2.83)	0.03	2.72<br>(2.59 to 2.86)	2.86<br>(2.71 to 3.00)	0.03	2.74<br>(2.63 to 2.85)	2.99<br>(2.79 to 3.19)	0.62	2.70<br>(2.54 to 2.86)	2.85<br>(2.73 to 2.98)	0.46	2.69<br>(2.57 to 2.8)	3.08<br>(2.88 to 3.29)	0.35
MID	2.97<br>(2.83 to 3.11)	2.75<br>(2.62 to 2.89)		2.91<br>(2.77 to 3.05)	2.81<br>(2.67 to 2.94)		2.82<br>(2.69 to 2.96)	2.89<br>(2.75 to 3.04)		2.80<br>(2.69 to 2.91)	3.07<br>(2.87 to 3.26)		2.77<br>(2.62 to 2.93)	2.92<br>(2.79 to 3.04)		2.76<br>(2.65 to 2.87)	3.18<br>(2.98 to 3.38)	
SF	3.12<br>(2.99 to 3.26)	2.96<br>(2.82 to 3.09)		3.12<br>(2.98 to 3.26)	2.96<br>(2.82 to 3.09)		3.02<br>(2.89 to 3.15)	3.06<br>(2.92 to 3.20)		2.99<br>(2.88 to 3.10)	3.21<br>(3.01 to 3.41)		2.98<br>(2.82 to 3.14)	3.08<br>(2.95 to 3.20)		2.97<br>(2.86 to 3.08)	3.29<br>(3.09 to 3.49)	
**Interstitial inflammation, %***																		
JM	2.07<br>(1.91 to 2.22)	1.65<br>(1.51 to 1.79)	0.70	1.85<br>(1.70 to 2.00)	1.86<br>(1.71 to 2.01)	0.61	1.80<br>(1.64 to 1.95)	1.91<br>(1.77 to 2.06)	0.19	1.82<br>(1.7 to 1.94)	1.99<br>(1.78 to 2.19)	0.51	1.81<br>(1.65 to 1.98)	1.88<br>(1.74 to 2.02)	0.42	1.83<br>(1.70 to 1.95)	2.04<br>(1.80 to 2.28)	0.52
MID	2.12<br>(1.96 to 2.27)	1.74<br>(1.60 to 1.87)		1.95<br>(1.80 to 2.10)	1.89<br>(1.74 to 2.04)		1.86<br>(1.70 to 2.01)	1.98<br>(1.84 to 2.13)		1.89<br>(1.77 to 2.01)	2.05<br>(1.85 to 2.25)		1.88<br>(1.72 to 2.05)	1.95<br>(1.81 to 2.09)		1.87<br>(1.75 to 1.99)	2.12<br>(1.88 to 2.35)	
SF	2.32<br>(2.17 to 2.48)	2.04<br>(1.90 to 2.17)		2.2<br>(2.05 to 2.35)	2.16<br>(2.01 to 2.31)		2.19<br>(2.04 to 2.35)	2.16<br>(2.02 to 2.31)		2.14<br>(2.02 to 2.26)	2.33<br>(2.13 to 2.53)		2.14<br>(1.98 to 2.3)	2.20<br>(2.06 to 2.34)		2.16<br>(2.03 to 2.28)	2.37<br>(2.13 to 2.61)	
**Arterial intima-media ratio ***																		
JM	0.06<br>(-0.01 to 0.13)	0.11<br>(0.05 to 0.17)	0.46	0.12<br>(0.05 to 0.18)	0.06<br>(-0.01 to 0.12)	0.96	0.06<br>(0.00 to 0.12)	0.12<br>(0.05 to 0.18)	0.65	0.07<br>(0.02 to 0.13)	0.14<br>(0.05 to 0.24)	0.47	0.08<br>(0.01 to 0.15)	0.09<br>(0.03 to 0.15)	0.51	0.08<br>(0.02 to 0.14)	0.05<br>(-0.05 to 0.15)	0.35
MID	-0.13<br>(-0.20 to -0.07)	-0.05<br>(-0.11 to 0.01)		-0.08<br>(-0.15 to -0.01)	-0.10<br>(-0.17 to -0.04)		-0.13<br>(-0.2 to -0.07)	-0.05<br>(-0.12 to 0.02)		-0.1<br>(-0.15 to -0.04)	-0.08<br>(-0.17 to 0.02)		-0.1<br>(-0.17 to -0.03)	-0.08<br>(-0.15 to -0.02)		-0.13<br>(-0.18 to -0.07)	-0.09<br>(-0.20 to 0.01)	
SF	-0.32<br>(-0.39 to -0.25)	-0.20<br>(-0.26 to -0.14)		-0.22<br>(-0.28 to -0.15)	-0.30<br>(-0.37 to -0.24)		-0.34<br>(-0.41 to -0.28)	-0.17<br>(-0.24 to -0.11)		-0.26<br>(-0.32 to -0.21)	-0.24<br>(-0.33 to -0.14)		-0.31<br>(-0.38 to -0.24)	-0.22<br>(-0.28 to -0.16)		-0.28<br>(-0.33 to -0.22)	-0.23<br>(-0.33 to -0.13)	
**Arteriolar hyalinosis index**																		
JM	0.90<br>(0.84 to 0.95)	0.70<br>(0.65 to 0.75)	0.15	0.81<br>(0.75 to 0.86)	0.78<br>(0.73 to 0.83)	0.52	0.74<br>(0.69 to 0.79)	0.85<br>(0.79 to 0.9)	0.27	0.81<br>(0.77 to 0.85)	0.73<br>(0.65 to 0.81)	0.35	0.79<br>(0.73 to 0.85)	0.80<br>(0.74 to 0.85)	0.72	0.73<br>(0.68 to 0.77)	0.95<br>(0.88 to 1.03)	0.30

MID	0.81 (0.75 to 0.86)	0.60 (0.55 to 0.65)	0.70 (0.65 to 0.76)	0.70 (0.64 to 0.75)	0.66 (0.61 to 0.71)	0.74 (0.69 to 0.8)	0.70 (0.66 to 0.75)	0.69 (0.61 to 0.76)	0.71 (0.65 to 0.77)	0.69 (0.64 to 0.75)	0.65 (0.60 to 0.70)	0.82 (0.74 to 0.90)
SF	0.77 (0.72 to 0.82)	0.55 (0.50 to 0.60)	0.67 (0.61 to 0.73)	0.64 (0.59 to 0.70)	0.61 (0.55 to 0.66)	0.71 (0.65 to 0.76)	0.66 (0.61 to 0.70)	0.65 (0.58 to 0.73)	0.64 (0.59 to 0.70)	0.67 (0.61 to 0.72)	0.61 (0.56 to 0.65)	0.80 (0.72 to 0.88)

* Value is log transformed.
Adjusted mean values is age- and sex- adjusted.
Abbreviations: IFTA, interstitial fibrosis/tubular atrophy; JM, juxtamedullary cortex; MID, middle cortex; SF, superficial cortex.

Table S4. Association between CKD stage and the presence of pathological findings defined based on each cutoff

	eGFR≥60 mL/min/1.73m^2	eGFR 45 to <60 mL/min/1.73m^2	eGFR <60 mL/min/1.73m^2	P for trend
**IFTA**				
With IFTA of ≥5%	102 (99.0)	37 (100)	32 (100)	0.23
With IFTA of ≥25%	25 (24.3)	13 (35.1)	19 (59.4)	<0.001
With IFTA of ≥50%	1 (1.0)	3 (8.1)	4 (12.5)	0.002
**Interstitial inflammation**				
With interstitial inflammation of ≥5%	24 (23.3)	13 (35.1)	16 (50.0)	0.04
With interstitial inflammation of ≥25%	2 (1.9)	2 (5.4)	2 (6.2)	0.09
With interstitial inflammation of ≥50%	0	0	1 (3.1)	0.04

Abbreviations: CKD, chronic kidney disease; eGFR, estimated glomerular filtration rate; IFTA, interstitial fibrosis/tubular atrophy.
Cochran–Armitage test

Table S5. Association between kidney cortical regions and the presence of pathological findings defined based on each cutoff

	JM cortex	MID cortex	SF cortex	P for trend
**IFTA**				
With IFTA of ≥5%	168 (97.7)	168 (97.7)	170 (98.8)	NA
With IFTA of ≥25%	49 (28.5)	58 (33.7)	77 (44.8)	<0.001
With IFTA of ≥50%	1 (5.8)	7 (4.1)	14 (8.1)	0.23
**Interstitial inflammation**				
With interstitial inflammation of ≥5%	112 (65.1)	125 (72.7)	145 (84.3)	<0.001
With interstitial inflammation of ≥25%	6 (3.5)	7 (4.1)	12 (7.0)	0.17
With interstitial inflammation of ≥50%	99 (96.1)	168 (97.7)	170 (98.8)	NA

Abbreviations: IFTA, interstitial fibrosis/tubular atrophy; JM, juxtamedullary cortex; MID, middle cortex; SF, superficial cortex.
Cochran–Armitage test

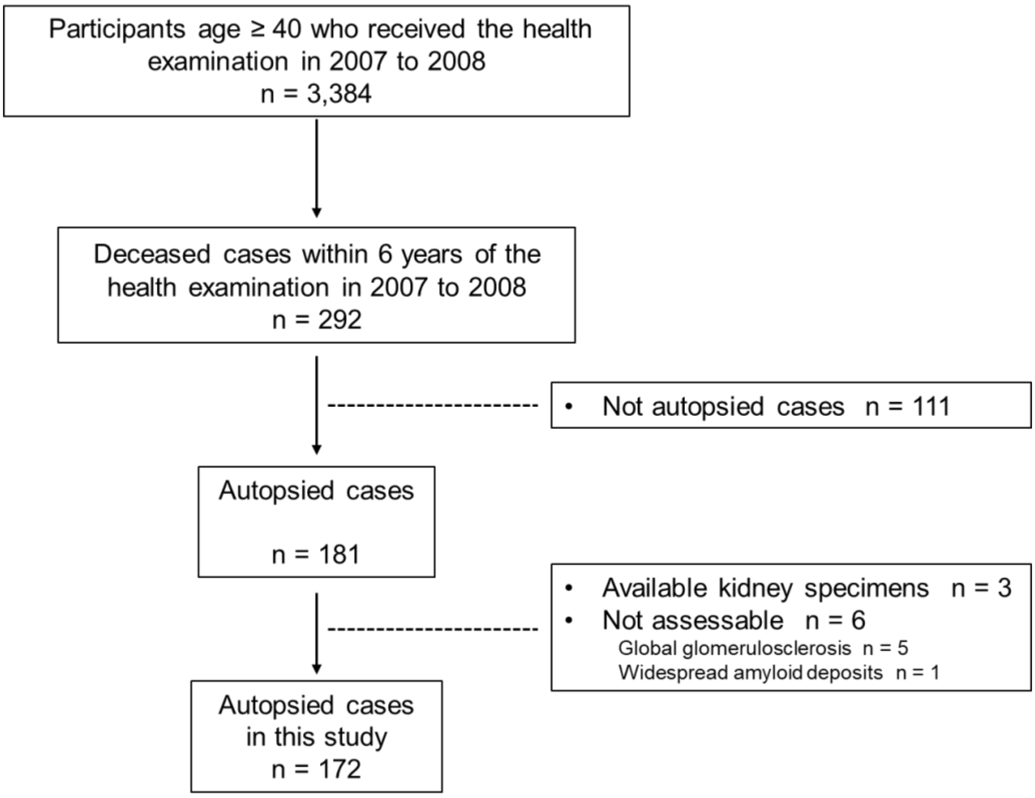

Figure S1. Selection flowchart of autopsied cases.

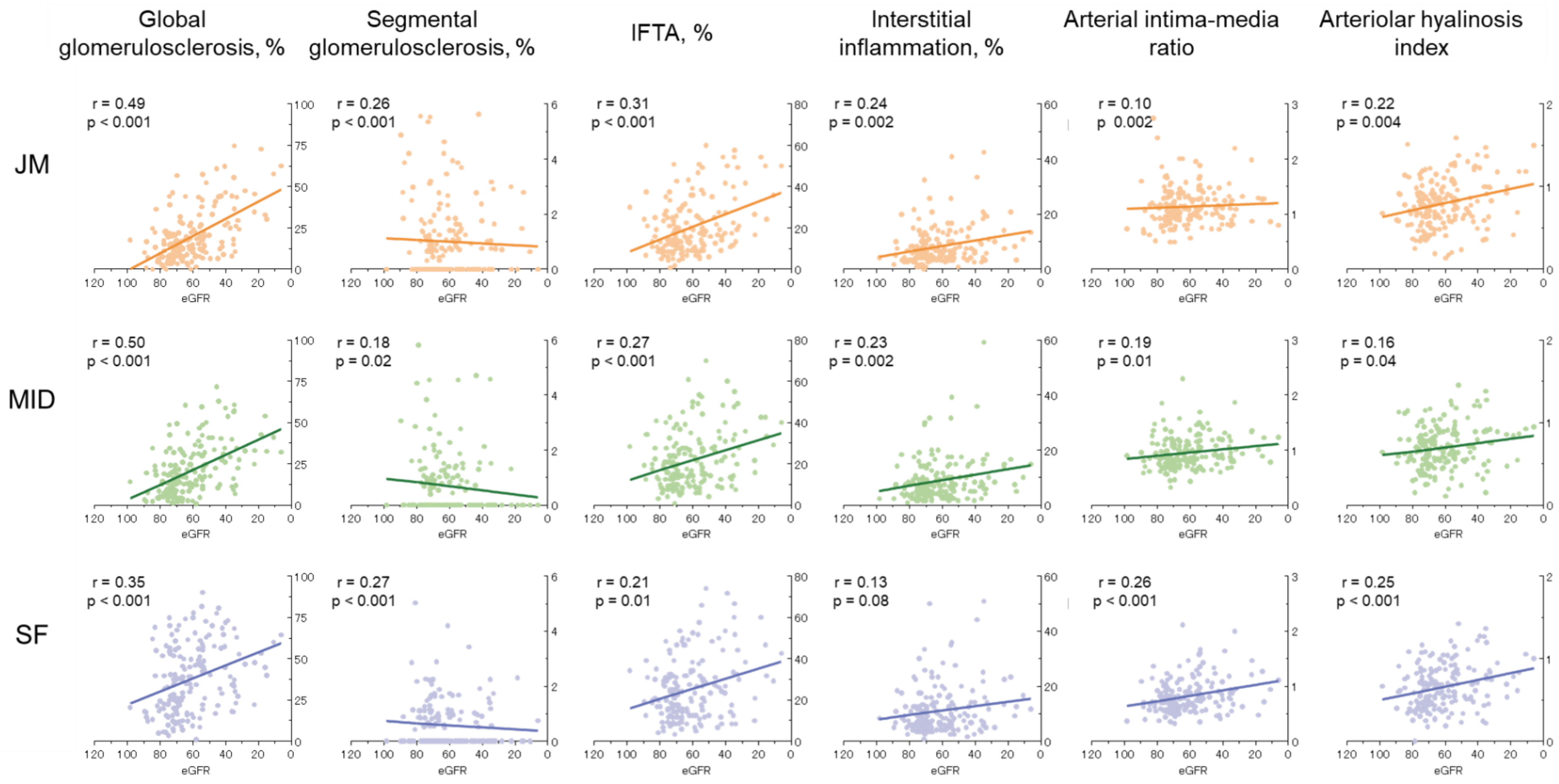

Figure S2. Correlation between histopathological findings and eGFR by cortical region (n = 172).

Abbreviations: eGFR, estimated glomerular filtration rate; JM, juxtamedullary cortex; MID, middle cortex; SF, superficial cortex
